# An Electrochemical Sensor Based on Carbon Paper Modified with Graphite Powder for Sensitive Determination of Sunset Yellow and Tartrazine in Drinks

**DOI:** 10.3390/s22114092

**Published:** 2022-05-27

**Authors:** Natalia Yu. Stozhko, Ekaterina I. Khamzina, Maria A. Bukharinova, Aleksey V. Tarasov

**Affiliations:** 1 Department of Physics and Chemistry, Ural State University of Economics, 8 Marta St. 62, 620144 Yekaterinburg, Russia; xei260296@mail.ru; 2 Scientific and Innovation Center of Sensor Technologies, Ural State University of Economics, 8 Marta St. 62, 620144 Yekaterinburg, Russia; m.a.buharinova@usue.ru (M.A.B.); tarasov_a.v@bk.ru (A.V.T.)

**Keywords:** food colorants, Sunset Yellow, Tartrazine, carbon paper, carbon veil, electrochemical sensor, modified electrode, graphite powder, voltammetry, soft and alcoholic drinks

## Abstract

The paper describes the development of an electrochemical sensor to be used for the determination of synthetic food colorants such as Sunset Yellow FCF (SY) and Tartrazine (TZ). The sensor is a carbon paper (CP) electrode, manufactured by using hot lamination technology and volume modified with fine-grained graphite powder (GrP). The sensor (GrP/CP) was characterized by scanning electron microscopy, energy dispersive spectrometry, electrochemical impedance analysis, cyclic, linear sweep and differential pulse voltammetry. The mechanism of SY and TZ electrochemical oxidation on GrP/CP was studied. The developed sensor has good electron transfer characteristics and low electron resistance, high sensitivity and selectivity. Applying the differential pulse mode, linear dynamic ranges of 0.005–1.0 μM and 0.02–7.5 μM with limits of detection of 0.78 nM and 8.2 nM for SY and TZ, respectively, were obtained. The sensor was used to detect SY and TZ in non-alcoholic and alcoholic drinks. The results obtained from drink analysis prove good reproducibility (RSD ≤ 0.072) and accuracy (recovery 96–104%).

## 1. Introduction

Naturally occurring and synthetic colorants are widely used in the food industry to make food stuffs look more delightful, appealing and healthy. Most colorants derived from natural origins are unstable and easily destroyed during food processing. In comparison with natural food colorants, synthetic ones seem to have higher stability and resistance to heating and ultraviolet radiation, changes in the pH of the medium, and effects of acids and alkali. In the food industry, synthetic colorants can be used both as individual compounds and in a mixture, which enables the obtaining of colors and shades that cannot be created with a single pigment.

Azo colorants constitute the largest group of synthetic food dyes. They account for 70% of all organic colorants produced worldwide [1]. Toxicological studies show that azo colorants can have adverse health effects on the human body by causing diarrhea, eczema, allergic reactions and asthma attacks [2], cancer and low levels of dopamine in the human brain [3,4]. Primarily, this is due to the fact that the products of azo colorant decomposition (e.g., aromatic amines) are toxic and carcinogenic [5]. Some studies prove that azo colorants can interact with human serum albumin [6] and hemoglobin [7]. Excessive use of artificial food dyes in children’s diets may cause hyperactivity as the most common behavior disorder [8]. Sunset Yellow FCF (E110) and Tartrazine (E102) are among the most widely used—individually as well as in combination—coloring agents for such food commodities as confectionery, sweets, and beverages. The accepted daily intake (ADI) of Sunset Yellow (SY), set by the World Health Organization (WHO) and Food and Agriculture Organization (FAO), is 4 mg kg^−1^ body weight per day [9], and the ADI of Tartrazine (TZ) is 10 mg kg^−1^ body weight per day [10]. The maximum permitted levels of SY in non-alcoholic/alcoholic drinks are 50/200 mg L^−1^ [9]. The maximum permitted levels of TZ in non-alcoholic/alcoholic drinks are 100/200 mg L^−1^ [10]. To ensure food safety, it is important to control the content of synthetic azo colorants, which poses researchers with the task of developing simple, low-cost, express and reliable methods for determining food dyes.

There are several analytical methods that were reported for the separation, identification and determination of food colorants, namely: spectrophotometry [11,12,13], chromatography (liquid and thin-layer) [14,15,16] and capillary electrophoresis [17,18]. Despite the advantages of these methods, they have some shortcomings. Spectroscopic methods have low selectivity, since food additives contain different functional groups that absorb or emit light, and, therefore, cannot always be correctly identified with the use of spectroscopic methods. Chromatographic methods and capillary electrophoresis require complex pre-processing of food samples, expensive equipment, and highly qualified staff.

Electrochemical methods present a credible and attractive alternative to the above methods since they have high sensitivity and selectivity for the determination of food dyes; they are simple, accurate and express, robust enough, require inexpensive, compact and portable equipment [19,20], are capable of being performing “on site” and with “in situ” analysis. The simplicity of sample preparation and the low consumption of reagents and samples due to the miniaturization of the measuring cell are additional benefits that make electroanalysis economically feasible [21].

To determine food azo colorants, cathodic and anodic voltammetry is used, since in their molecular structure azo colorants contain a chromophore azo group (–N=N–), capable of electrochemical reduction, and a hydroxyl OH^−^ group on the aromatic ring capable of oxidation. When determining food azo dyes, TZ and SY in particular, the following electrodes are used: a glassy carbon electrode (GCE) [22]; boron-doped diamond electrode [17]; a carbon paste electrode (CPE) [23,24]; and a screen-printed carbon electrode (SPCE) [25]. To improve sensitivity and selectivity of the determination of TZ and SY, the electrodes are modified with various nanomaterials: carbon nanotubes [26], graphene [27], metal nanoparticles (Au, Ag) [28,29,30] and oxides (TiO_2_, NiO, ZnO, Dy_2_O_3_, Fe_3_O_4_, SiO_2_) [31,32,33,34] and different combinations of these modifiers [35,36,37]. These modifiers contribute to an increase in the electroactive area of the electrode surface, the appearance of adsorption accumulation centers, a decrease in the so-called “chemical noises” caused by impurity discharge currents and charging current [17], and acceleration of the kinetics of the electrode process. The material and structure of the electrode surface may have a significant impact on the fixation of the modifier and the properties of the modified electrode as a whole. To improve the adhesion of the modifier to the electrode surface, it is mechanically cleaned using abrasive materials [38] and then is electrochemically activated [39,40]. These operations are time-consuming and can often cause non-reproducibility of the surface structure and measurement results. This problem makes the search for and use of new electrode materials quite relevant.

In recent years, carbon paper (CP) (or carbon veil) has been used as an electrochemical interface [41,42,43]. This material incorporates porous carbon fibers that bond together and form a three-dimensional (3D) structure with high electrical conductivity, specific surface area, and flexibility. Unlike a conventional flat surface, a three-dimensional matrix of carbon fibers creates a more rigid fixation of the modifier throughout the CP, can significantly enlarge the surface area during modification and provides a short diffusion for electrons and ions during detection [44]. CP is suitable for mass and cheap production of electrodes. When used in electroanalysis, CP is modified with nanoparticles of metals and bimetals (Ni, Ag, Au, Pt, Au–Sn), carbon nanotubes, and graphene [45]. CP sensors are known to be used for the determination of glucose [41,42], dopamine, nitrites [43,46], uric [47] and ascorbic acids [48]. However, no CP sensors for the determination of azo colorants have been developed yet.

The purpose of this work was to develop a highly sensitive, selective, simple and cheap CP-based sensor for the determination of TZ and SY. To achieve this goal, the following objectives were set:

- To select a CP modifier,
- To specify optimal conditions and study the mechanism of electrochemical transformation of azo colorants on a modified CP electrode,
- To evaluate the analytical capabilities of the developed sensor,
- To test the developed sensor in the electroanalysis of beverages.

## 2. Materials and Methods

### 2.1. Chemicals and Materials

Sunset Yellow FCF (E110) and Tartrazine (E102) were purchased from Merck KGaA (Darmstadt, Germany). Na_2_HPO_4_·12H_2_O (JSC Vekton, St. Petersburg, Russia), KH_2_PO_4_ (NevaReaktiv Ltd., St. Petersburg, Russia), acetone (JSC Ecos-1, Moscow, Russia), KCl (Ecros-Analytica Ltd., St. Petersburg, Russia), K_3_[Fe(CN)_6_]·3H_2_O (AO Reachem Ltd., Moscow, Russia), urea (Fluka Chemie GmbH, Buchs, Switzerland), ascorbic acid and taurine (Sigma-Aldrich Co., St. Louis, MO, USA), caffeine (Sigma-Aldrich, Chemie GmbH, Steinheim, Germany), glucose and calcium chloride (NevaReaktiv Ltd., St. Petersburg, Russia), tartaric acid (Merck KGaA, Darmstadt, Germany), ammonium chloride, sodium carbonate, and sodium citrate (JSC ChemReactivSnab, Ufa, Russia). All chemicals were used without further purification. Ultrapure water with a resistivity of 18 MΩ cm was used as the solvent.

Working electrodes were manufactured from carbon paper (veil) (M-Carbo Ltd., Minsk, Belarus), attached to polyethylene terephthalate film sized 303 × 216 × 0.125 mm (Fellowes Inc., Itasca, IL, USA). Electrodes were modified with carbon nanomaterials: graphite powder (Aldrich Chemical Company, Inc., Milwaukee, WI, USA), multi-walled carbon nanotubes (Sigma-Aldrich Co., LLC, St. Louis, MO, USA), carbon black N220 (Cabot Corporation, Ravenna, Italy), graphene RG-S1 (Rusgraphen Ltd., Moscow, Russia).

### 2.2. Equipment and Electrodes

A laminator machine LM-260iD (Rayson Electrical MFG, Ltd., Foshan, China) was used to manufacture CP electrodes.

Microscopic studies of the surface of the modified electrodes were carried out on a Carl Zeiss Evo Ma 15 scanning electron microscope (Carl Zeiss Industrielle Messtechnik GmbH, Oberkochen, Germany) equipped with an X-Max^N^ 80 energy dispersion spectrometer with silicone drift detector (Oxford Instruments Analytical Ltd., High Wycombe, UK). SEM images of the surface of bare and modified electrodes were obtained under conditions of accelerating voltage—20 kV.

A semi-automatic computerized voltammetric analyzer IVA-5 (IVA, Ltd., Yekaterinburg, Russia) with a PE-6100 magnetic stirrer and a three-electrode cell was used for cyclic and linear sweep voltammetric studies. Differential pulse studies and electrochemical impedance spectroscopy (EIS) measurements were carried out on µAutolab Type III potentiostat/galvanostat (Metrohm, Herisau, Switzerland). An electrochemical cell included a CP working electrode and a modified CP electrode; a reference electrode EVL-1M3.1 (Ag/AgCl/KCl, 3.5 M) (JSC Gomel Plant of Measuring Devices, Gomel, Belarus) and an auxiliary electrode, a carbon rod.

To monitor pH of background solutions a pH/ions-meter TA-Ion (RPE Tomanalyt Ltd., Tomsk, Russia) was used. An ultrasonic bath RH PS-40A (Shenzhen Codyson Electrical Co., Ltd., Guangdong, China) was used to prepare suspensions of graphite modifiers. Akvalab-UVOI-MF-1812 installation (JSC RPC Mediana-Filter, Moscow, Russia) was used to obtain ultrapure water.

### 2.3. Procedures

#### 2.3.1. Electrodes Preparation

To prepare the electrodes, a fast and simple hot lamination technology was used [46,48]. For this purpose, CP was fixed on a polyethylene terephthalate substrate with an adhesive layer by rolling through laminator rolls heated to 140 °C. The resulting piece was cut into separate electrodes. The working and contact zones of the CP electrode were separated by applying an insulating layer (cementite: acetone, v:v = 1:5) to the middle zone of the electrode. Thus, the geometric area of the working zone is 12 mm^2^. Further, the working area of the electrode was modified with various materials using the drop-casting method. For modification 3, 5, 10, 15, 20, 25, 30 µL of carbon modifiers suspension with a concentration of 5 mg mL^−1^ were taken. Carbon modifiers were prepared by mixing a sample of the material with a solvent (H_2_O: acetone, v:v = 9:1) and further ultrasonic treatment for 15 min.

#### 2.3.2. Electrochemical Measurements

Cyclic voltammograms in the presence of SY и TZ were recorded in the range from (0.1) 0.3 V to 1.5 V in the phosphate buffer solution pH 5. Cyclic voltammograms of K_3_[Fe(CN)_6_] were registered from −0.4 V to +1.1 V. Linear sweep voltammograms were recorded in the range of potentials from 0.3 V to 1.3 V while studying the effect of pH of the supporting electrolyte and the potential scan rate on electrooxidation of SY and TZ. The accumulation potential of colorants was 0.0 V in stripping voltammetry. Differential pulse voltammograms of SY and TZ were obtained from 0.3 V to 1.3 V applying the potential scan rate 50 mVs^−1^, modulation amplitude 80 mV, step potential 15 mV, and modulation time 120 ms. EIS measurements were performed in the presence of 0.01 M equimolar mixture K_3_[Fe(CN)_6_]/K_4_[Fe(CN)_6_] in 0.1 M KCl at the working potential 0.2 V in the range of frequency from 0.1 Hz to 100 kHz.

#### 2.3.3. Sample Preparation

The samples of alcoholic and non-alcoholic drinks produced in the Russian Federation were purchased from local supermarkets in Ekaterinburg (Russia). The analysis was carried out without additional sample preparation and degasation of the samples. Both 50- and 100-fold dilutions of the samples were used for analysis. Parallel measurements of the same beverage sample were carried out using a new GrP/CP electrode and freshly prepared solutions each time.

#### 2.3.4. Calculations and Result/Data Processing

The measurement data were statistically analyzed for five parallels with the confidence level P = 0.95. The results are presented as average value ± confidence interval.

The signal-to-noise ratio (SNR) was measured following Equation (1) [49]

(1) $$\mathrm{SNR}=\frac{h}{S_{N}}$$

where h is the height of the peak, S_N_ is standard deviation of the noise.

Limits of detection (LOD) were calculated using Equation (2):

(2) $$\mathrm{LOD}=\frac{3\sigma }{b}$$

where σ is the standard deviation of analytical colorant signals corresponding to minimal concentrations (5.0 nM of SY and 20 nM of TZ); b is the slope of dependence I_pa_ = f(C_dye_). The content of SY and TZ in the real samples was measured by the standard addition method under the optimal detection conditions.

Accuracy was determined through recovery experiments. Recovery was calculated using Equation (3) in accordance with the IUPAC Recommendations

(3) $$\mathrm{Recovery}\left(\%\right)=\frac{Q_{A}\left(O+S\right)-Q_{A}\left(O\right)}{Q_{A}\left(S\right)}\times 100,$$

where Q_A_(S) is the quantity of analyte A added (spike value) and Q_A_(O + S) is the quantity of A recovered from the spiked sample and Q_A_(O) from the original sample [50].

## 3. Results

**Figure 1:**
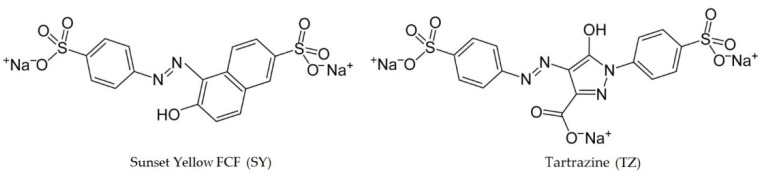
Structural formulas of SY and TZ.

According to the literature, the analytical signals of most azo colorants are of an adsorption nature [26,27,30]. Hence, in order to obtain high analytical characteristics of azo colorant determination, it is important to have electrodes whose surface possesses good sorption properties. A variety of carbon micro- and nanostructures, due to their high specific surface area and capability to demonstrate different intermolecular interactions, opens up broad prospects for their use in analytical chemistry and sorption technologies [51]. Azo dye molecules can interact with carbon micro- and nanoparticles by building π–π bonds between the delocalized system of π-electrons of carbon nanomaterial and phenyl groups of organic compounds (Figure 1), the electrostatic attraction between colorant ions and carbon hexagons, and hydrophobic interactions between compounds, containing condensed benzene rings.

This type of interaction can ensure effective adsorption concentration of azo colorants on a carbon-containing surface, therefore, it was reasonable to use up-to-date carbon materials as modifiers of the CP electrode, such as multi-walled carbon nanotubes (MWCNTs), carbon black (CB), graphene plates (GR) and graphite powder (GrP). All these carbon materials have a small size, large specific surface area, resistance to destruction, high-temperature resistance and thermal conductivity, low electrical resistance, sufficiently high electrocatalytic activity, and good adsorption properties [52]. More specific characteristics of carbon modifiers are given in Table 1.

### 3.1. Electronic Microscopy of Electrodes

**Figure 2:**
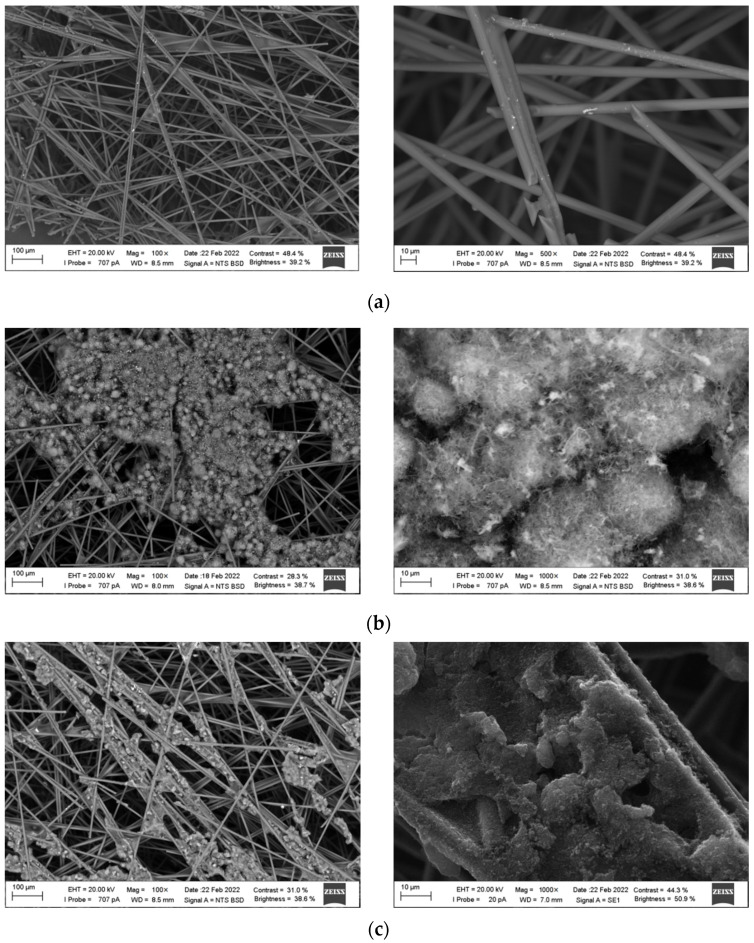

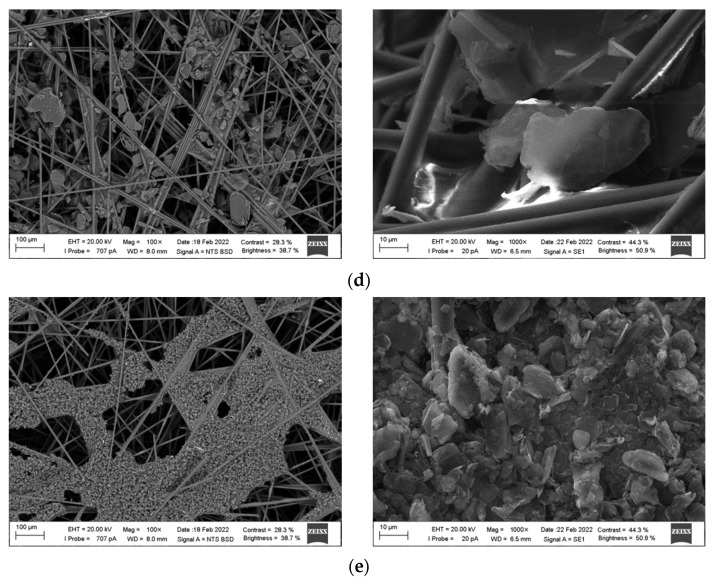
SEM images of the bare CP (**a**) and the CP modified with different carbon materials: MWCNTs (**b**), CB (**c**), GR (**d**) and GrP (**e**).

Figure 2 presents SEM images of the bare CP and the CP modified with different modern carbon materials. As can be seen from Figure 2, the modification of CP with MWCNTs, CB, GR, and GrP results in the formation of different surface layers. A common point for all modified CP electrodes is the fact that none of the carbon materials forms a uniform coating on their surfaces, since carbon-containing particles fall through between the fibers due to the mesh structure of some parts of the CP surface, which leads to volumetric modification of the electrode. MWCNTs form sufficiently large agglomerates in the shape of loosely clumped tangles of 5–40 µm in size on the CP surface. CB forms smaller than MWCNTs aggregates and they are located mainly along the CP fibers. Thin GRs modify CP throughout the entire volume, with larger retainers located in the upper layers of CP, and smaller ones in the lower layers. GrP microparticles form the finest-grained structure with a well-developed surface. Thus, different electrochemical behavior of SY and TZ might be expected on modified CP electrodes due to the difference in the structure of their surfaces.

### 3.2. Electrochemical Behavior of SY and TZ on Modified CP Electrodes

**Figure 3:**
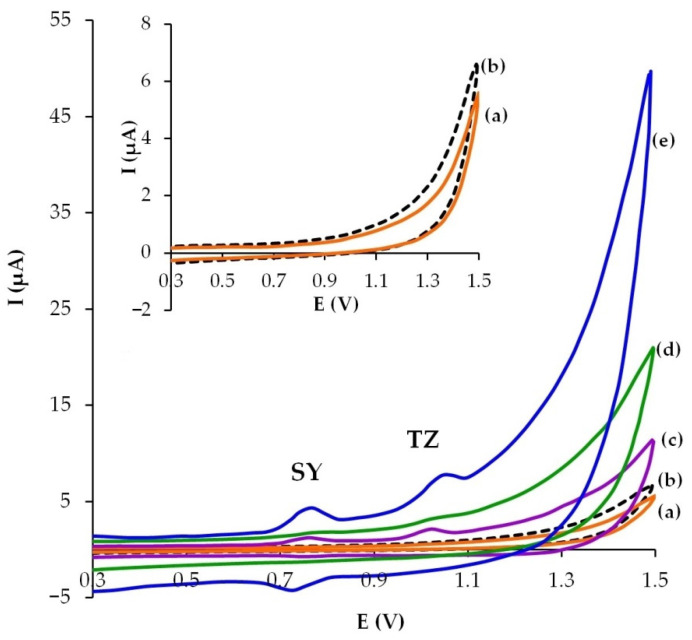
Cyclic voltammograms of 10 μM SY and TZ on the bare CP electrode (**b**) and the CP electrodes modified with GR (**a**), MWCNTs (**c**), CB (**d**), GrP (**e**). Supporting electrolyte: phosphate buffer solution pH 5, ν = 50 mV s^−1^.

Figure 3 shows cyclic voltammograms of SY and TZ obtained using the bare CP electrode and the CP electrodes modified with carbon nano- and micro-materials. As can be seen from Figure 3, on the bare (curve b) and the GR-modified CP electrodes (curve a), signals for 10 μM of SY and TZ are registered neither on the anodic nor cathodic branches of the cyclic voltammograms in phosphate buffer solution. When using electrodes modified with MWCNTs (curve c), CB (curve d) and GrP (curve e), two signals appear on the anode branch of the cyclic voltammogram at 0.77 V and 1.07 V, corresponding to the electrochemical oxidation of SY and TZ, respectively. On the cathodic branch, only one signal appears at 0.72 V, which corresponds to the electrochemical reduction of SY oxidation products, while no cathodic signal for TZ is observed. An increase in the colorant signals occurs in series of modified electrodes: CB/CP electrode < MWCNTs/CP electrode < GrP/CP electrode.

The signal-to-noise ratio (SNR) was used to characterize the modified electrodes that exhibited SY and TZ signals on cyclic voltammograms. The SNR for the SY signal was 21.6 for the GrP/CP electrode; 18.5 for the MWCNTs/CP electrode and 8 for the CB/CP electrode. The SNR for the TZ signal was 20, 18, and 9, respectively. The SNR decreased in the series GrP/CP > MWCNTs/CP > CB/CP electrodes.

The GrP/CP electrode, where the strongest signals for SY and TZ and SNR were observed, was used in further experiments.

### 3.3. Selection of the Optimum Volume of Modifier

The effect of the amount of the modifier on the electrochemical activity of the modified electrode was measured through the dependence of the colorant anodic peak current (I_pa_) on the quantity of graphite powder immobilized on the CP electrode.

**Figure 4:**
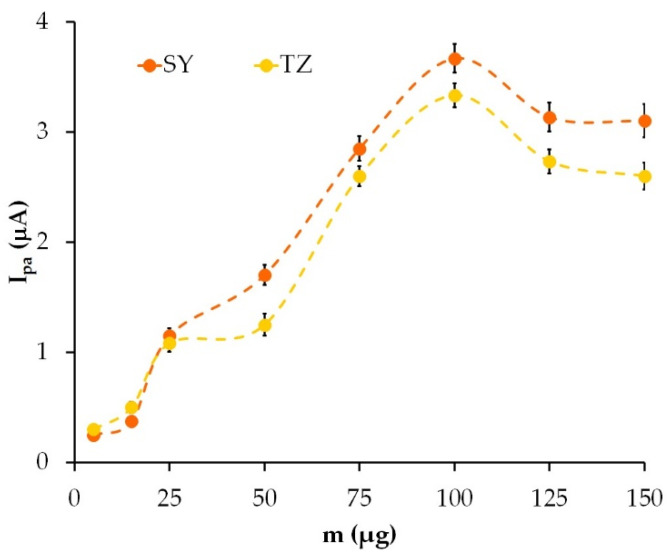
Dependence of oxidation peak current of 10 µM SY and TZ on the quantity of graphite powder immobilized on the CP electrode.

As can be seen from Figure 4, the strongest SY and TZ signals were obtained on CP electrodes, immobilized with 100 µg of graphite powder. With a graphite powder weight of more than 100 µg, a slight decrease in the colorant oxidation current was observed, which was caused by the detachment of loose carbon particles on the electrode surface. GrP(100 µg)/CP electrodes were used in further experiments.

### 3.4. Characteristics of CP and GrP/CP Electrodes

**Figure 5:**
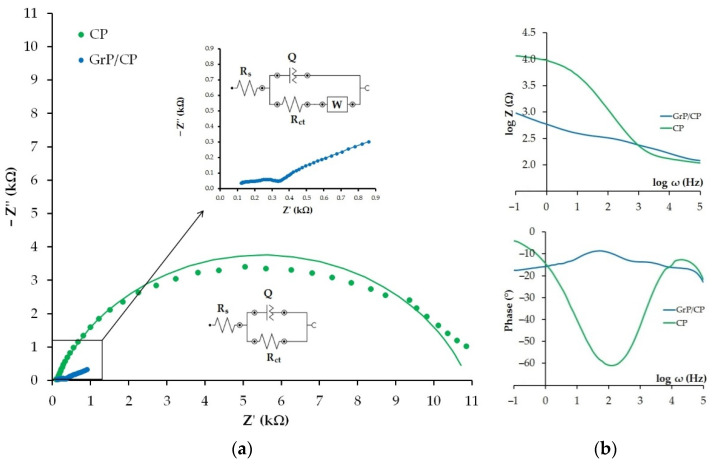
Experimental (points) and fitted (lines) Nyquist plots for CP and GrP/CP electrodes in 0.1 M KCl containing 0.01 M [Fe(CN)_6_]^3−/4−^ (**a**). Bode plots (**b**). Frequency (ω) range 0.1 Hz–100 kHz at polarization potential of 0.2 V. Inserts: Randles equivalent cells.

Properties of electron transfer on the bare and modified CP electrodes were carried out using the EIS method in 0.1 M KCl solution containing [Fe(CN)_6_]^3−/4−^. Figure 5 presents Nyquist plots for the CP and GrP/CP electrodes. EIS spectra of the CP electrode are in the shape of a semicircle, which indicates a constrained electron transfer. On the modified electrode, the shape of the curve has changed considerably: the semicircle has practically disappeared, but a straight line, with an approximately 45-degree slope, can be observed, which indicates on the diffusion limitation of the electrochemical process. EIS spectra were fitting using Randles equivalent cells consisting of electrolyte resistance (R_s_), constant phase element (Q), charge transfer resistance (R_ct_) and Warburg impedance (W). The Warburg element in the Randles circuit was used only for the GrP/CP electrode because the CP electrode spectra (Figure 5a, green curve) did not show the phenomenon of diffusion (straight at 45 degrees). R_ct_ for the GrP/CP electrode is 0.23 kOhm, which is massively smaller than for the CP electrode, whose resistance is 10.8 kOhm. An almost 50-times decrease in the semicircle diameter of the GrP/CP electrode as compared with the bare CP electrode indicates a dramatic decrease in the charge transfer resistance at the modified electrode. The constant phase element value for the GrP/CP electrode is 531 µOhm, which is 96.5-fold higher than for the bare CP with Q = 5.5 µOhm. The latter can be explained by an increase in electrode inhomogeneity and the electrode surface total charge due to the presence of GrP. The better conductive properties of the GrP/CP electrode in comparison with the bare CP electrode are supported by a Bode plot (Figure 5b) that expresses a log magnitude-phase frequency response. The GrP/CP electrode demonstrates lower impedance values in the range of frequencies from 0.1 to ~100 Hz (plot: log Z vs. log ω) as well as a narrower phase lag in the range from 9° to 18° depending on frequency (plot: Phase vs. log ω), as compared with the bare CP electrode whose phase lag changes from 5° to maximum 61° at 100 Hz.

Thus, the obtained data confirm better electron-transfer properties of the GrP/CP electrode in comparison to the bare CP, as well as the effectivity of graphite powder as the electrode surface modifier in the development of electrochemical sensors.

**Figure 6:**
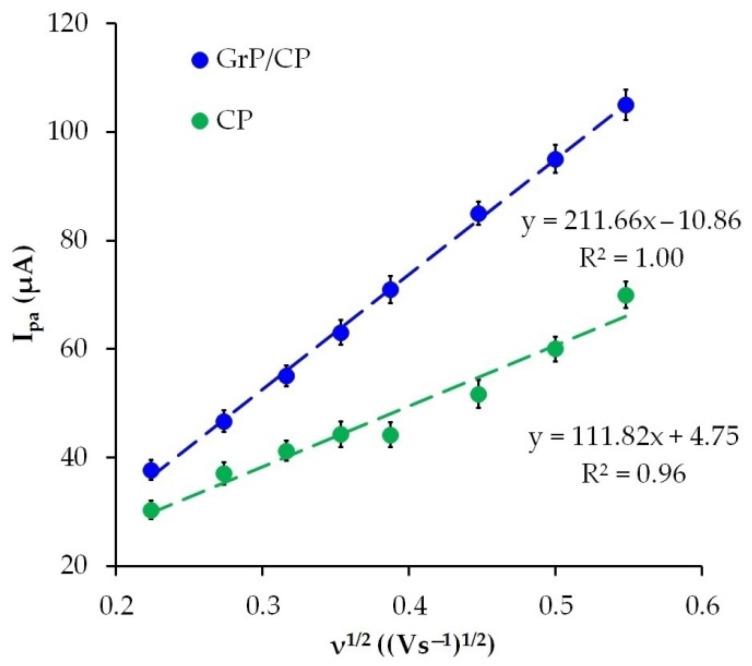
The plot of I_pa_ vs. ν^1/2^ for CP and GrP/CP electrodes.

To assess the effective surface area of the CP and GrP/CP electrodes, cyclic voltammograms were recorded for 1.0 mM K_3_[Fe(CN)_6_] in 0.1 M KCl at different potential scan rates (Figure S2). The dependence of I_pa_ vs. ν^1/2^ for the CP and GrP/CP electrodes was plotted (Figure 6). The electrode square was calculated using Randles–Sevcik Equation (4):

(4) $$I_{\mathrm{pa}}=2.69\times 10^{5}z^{3/2}{\mathrm{ACD}}^{1/2}\nu^{1/2}$$

where I_pa_ is anodic peak current; z is the number of electrons transferred (z = 1); A is the effective surface area of the electrode; D is the diffusion coefficient for K_3_[Fe(CN)_6_] (7.6 × 10^−6^ cm^2^s^−1^ [53]); C is the molar concentration of K_3_[Fe(CN)_6_] (10^−6^ mol cm^−3^); and ν is scan rate. The voltammogram slope I_pa_ vs. ν^1/2^ was used to calculate the electroactive surface area of the electrodes. The obtained data were 0.15 ± 0.01 for the CP electrode and 0.28 ± 0.03 cm^2^ for the GrP/CP electrode.

### 3.5. The pH Effect

**Figure 7:**
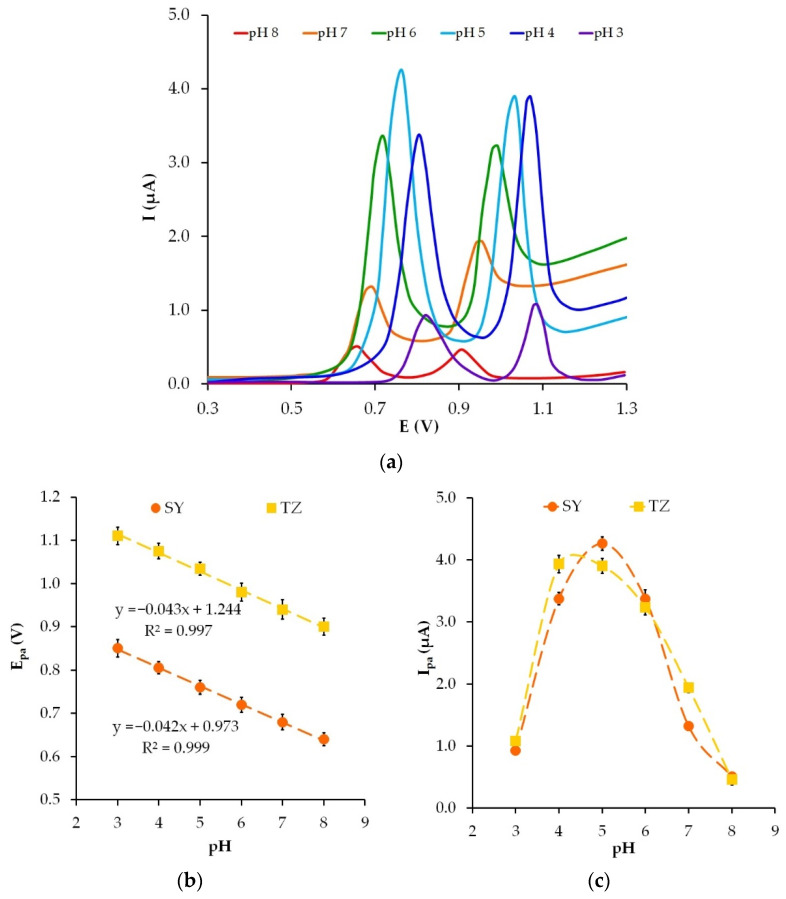
Linear sweep voltammograms of 10 μM SY and 10 μM TZ on GrP/CP electrode in phosphate buffer solution at different pH at the scan rate of 50 mV s^−1^ (**a**), the effects of pH on the anodic peak potentials (**b**) and anodic peak currents (**c**).

The pH of the supporting electrolyte is an essential parameter that affects the electrochemical oxidation of SY and TZ. The studies of the pH effect on the electrochemical oxidation of the colorants were carried out with the use of phosphate buffer solution in the pH range from 3 to 8. Linear sweep voltammograms at different pHs and the corresponding dependences I_p_ = f(pH) and E_p_ = f(pH) are illustrated in Figure 7.

There is a linear relationship between the anodic peak potential (E_pa_) of SY and TZ and the pH of the phosphate buffer solution. With an increase in pH, the E_pa_ of SY and TZ tends to shift in the negative direction (Figure 7b), which indicates the participation of protons in the electrochemical process. The dependence E_pa_ vs. pH is described by linear Equations (5) and (6):

E_pa_ (V) = −0.042 pH + 0.973 (R^2^ = 0.999) for SY (5)

E_pa_ (V) = −0.043 pH + 1.244 (R^2^ = 0.997) for TZ (6)

The slopes of E_pa_ = f(pH), 42 mV/pH for SY and 43 mV/pH for TZ, are close to theory Nernst value (59 mV/pH). Taking into account the slope of the obtained dependences and Equation (7), the ratio between the proton (m) and electrons (z) involved in the reaction was calculated as

(7) $$E_{\mathrm{pa}}=E^{0}-\frac{59m}{z}\mathrm{pH}$$

The m/z ratio, 0.71 for SY and 0.73 for TZ, indicates the equivalent number of protons and electrons involved in the electrooxidation of SY and TZ, which is justified in other studies [26,33,54].

The dependence of the oxidation current of SY and TZ on pH (I_pa_ = f(pH)), presented in Figure 7c, shows that the I_pa_ gradually increases with an increase in pH from 3 to 5. A change in pH from 5 to 8 causes a decrease in the current of colorant electrooxidation. The domed shape of the dependence I_pa_ = f(pH) may result from the impact of the medium acidity on the electrochemical oxidation of colorants [55]. It is known that SY and TZ are proton aromatic molecules that can be easily deprotonated with an increase in pH and can build various anionic forms [56]. The pKa values are 10.4 and 9.4 for TZ and SY, respectively [57]. In alkaline solutions, anionic forms of colorants predominate. Apparently, weak retention of colorant anions on the electrode surface by electrostatic attraction, as well as constrained electrode process in a proton-depleted medium, lead to a lower colorant oxidation current at pH > 5 [58].

A pH of 5 was chosen in further experiments as SY and TZ currents have the maximum value.

### 3.6. The Scan Rate Effect

**Figure 8:**
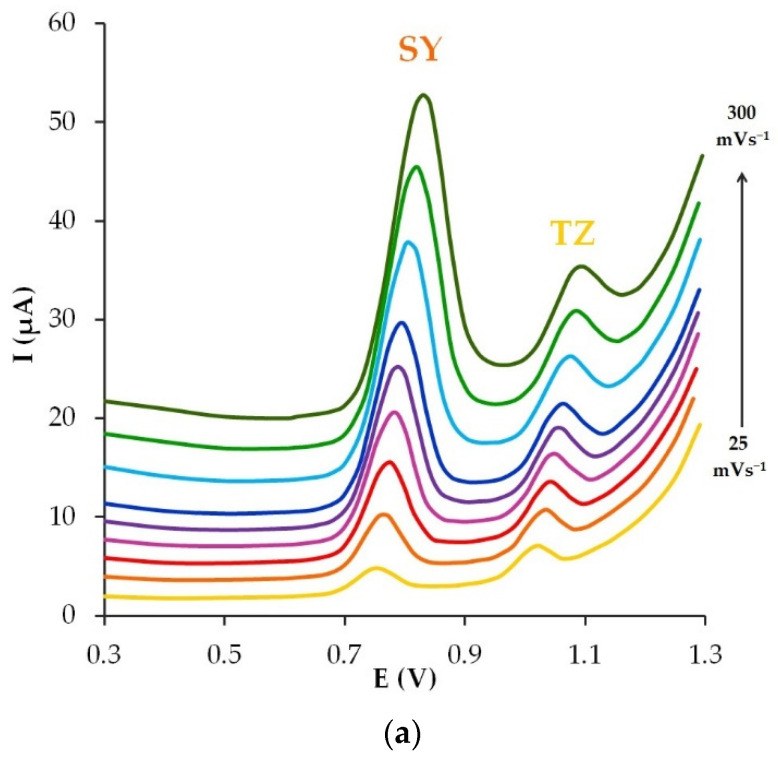

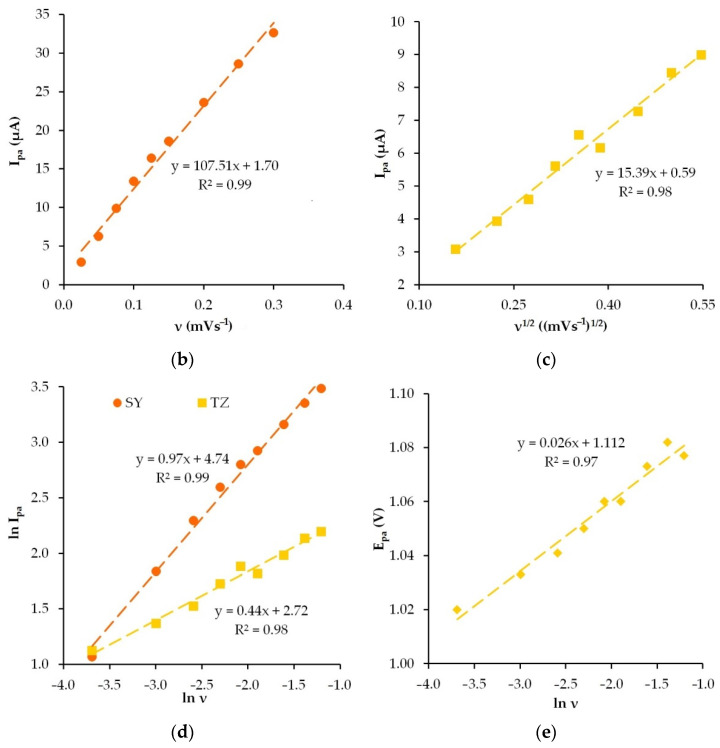
Linear sweep voltammograms of SY and TZ (10 µM each) on GrP/CP electrodein phosphate buffer solution pH 5 at different potential scan rates: 25, 50, 75, 100, 125, 150, 200, 250, 300 mV s^−1^ (**a**). Plots I_pa_ = f(ν) for SY (**b**), I_pa_ = f(ν^1/2^) for TZ (**c**) and ln I_pa_ = f(ln ν) (**d**), E_pa_ = f(ln ν) for TZ (**e**).

To understand the mechanism of the electroconversion process for SY and TZ, the electrochemical behavior of dyes on the GrP/CP electrode in phosphate buffer solution pH 5 at potential scan rates ranging from 25 mV s^−1^ to 300 mV s^−1^ was studied (Figure 8a).

As can be seen from Figure 8b, the I_pa_ of SY changes linearly depending on the scan rate in accordance with the linear regression Equation (8):

I_pa_(μA) = 107.51(mVs^−1^) + 1.7 (R^2^ = 0.99), (8)

which indicates a typical adsorption-controlled process. The anode peak current of TZ changes linearly when the values of the square root of the scan rate change according to the linear regression Equation (9):

I_pa_(µA) = 15.39(mV s^−1^)^1/2^ + 0.59 (R^2^ = 0.98), (9)

which indicates a typical diffusion-controlled process (Figure 8c).

To further confirm the adsorption- and diffusion-controlled electrochemical processes for SY and TZ, respective dependences between ln I_pa_ and ln ν were obtained (Figure 8d). These dependences are described by Equations (10) and (11) as follows:

lnI_pa_ (μA) = 0.97 ln ν (mV s^−1^) + 4.74 (R^2^ = 0.99) for SY, (10)

lnI_pa_(μA) = 0.44 ln ν (mV s^−1^) + 2.72 (R^2^ = 0.98) for TZ. (11)

A slope of the dependence of 0.97 in Equation (10) is close to 1, which confirms the adsorption-controlled nature of the electrode process for SY. The slope of 0.44 in Equation (11), close to 0.5, indicates the diffusion-controlled nature of the electrode process for TZ. These findings are consistent with the conclusions drawn from Figure 8b,c and the literature [33,59].

To calculate the number of electrons involved in the electrode process, it was assumed that the electrochemical transformation of SY is reversible, and of TZ—irreversible on the GrP/CP electrode. Indeed, in accordance with Figure 3, the potential difference (∆E) between the anodic and cathodic peaks of the SY current, which is 42 mV and close to 59 mV, and the ratio of these peak currents, equal to 0.98 and close to 1, confirm the reversible nature of SY electrooxidation. To calculate the number of electrons n involved in the SY electrooxidation, Equation (12) was used for a reversible electrode process:

(12) $$\Delta E=\frac{59}{z}.$$

Calculated z = 1.4 shows that one electron is involved in the electrical conversion of SY on the GrP/CP electrode.

The process of electrochemical transformation of TZ on the GrP/CP electrode is irreversible as there is no cathode signal of TZ on the CP electrode (Figure 3), therefore, Equation (13) was used to calculate the number of electrons:

(13) $$E_{p}=E^{0}+\frac{\mathrm{RT}}{\alpha \mathrm{zF}}\left[0.780+\ln\frac{D_{R}^{1/2}}{k^{0}}+\ln{\left(\frac{\alpha \mathrm{zF}\nu }{\mathrm{RT}}\right)}^{1/2}\right]=K+\frac{\mathrm{RT}}{2\alpha \mathrm{zF}}\ln\nu ,$$

where E^0^ is formal potential; α is the charge transfer coefficient; D is the diffusion coefficient; and k^0^ is the standard heterogeneous rate constant. Other symbols have their traditional meaning. Taking into account the slope of the linear dependence E_pa_ = f(ln v) (Figure 8e), equal to 0.0258, and α, equal to 0.5 for an irreversible electrode process, the calculated value of n was 0.91. Consequently, one electron participates in the TZ electrooxidation.

**Figure 9:**
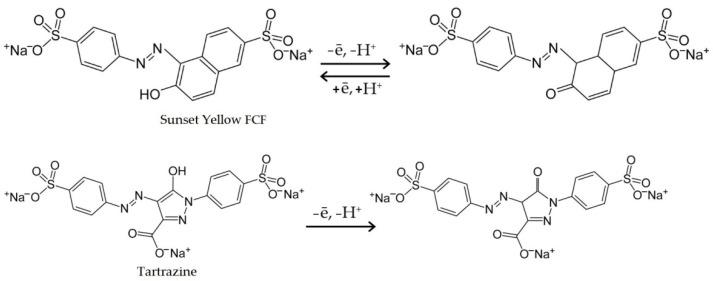
Electroconversion of SY and TZ.

Based on the results obtained and the literature, the diagram of the colorant electroconversion can be presented as follows (Figure 9):

### 3.7. Analytic Characteristics of the GrP/CP Electrode

Differential pulse voltammetry, known for its high sensitivity and resolution, was used to establish the analytical characteristics of the Gr/CP electrode. Optimal conditions, including the potential of accumulation (E_acc_) of the colorants on the GrP/CP electrode, as well as modulation amplitude, step potential and modulation time for registering differential pulse voltammograms, were selected based on the dependences of the electrochemical oxidation current of SY and TZ vs. these parameters (Supplementary Materials, Figure S3). The highest values of the colorant oxidation current were obtained under the following conditions:

- The potential of accumulation: 0–0.2 V for TZ and 0 mV for SY;
- The modulation amplitude: 70–80 mV for TZ and 80 mV for SY;
- The step potential: 12.5–20 mV for TZ and 15 mV for SY;
- The modulation time: 120 ms for TZ and SY.

A large difference between the peak potentials of several analytes allows for their simultaneous determination [60]. The separation value of the SY and TZ peaks is about 250 mV. This is enough so that the peak of SY does not interfere with the peak of TZ. Simultaneous accumulation and registration of SY and TZ were performed at the potential of accumulation 0 V, modulation amplitude 80 mV, step potential 15 mV and modulation time of 120 ms.

**Figure 10:**
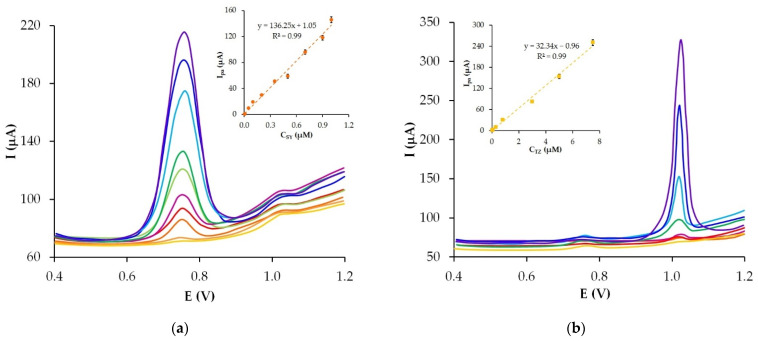
Differential pulse voltammograms of different SY concentrations (0.005, 0.01, 0.05, 0.1, 0.2, 0.35, 0.5, 0.7, 0.9, 1.0 µM) in the presence of 0.5 μM TZ (**a**) and different TZ concentrations (0.02, 0.04, 0.08, 0.3, 0.8, 3.0, 5.0, 7.5 µM) in the presence of 0.2 μM SY (**b**). Inserts: linear dependences of anodic peak current vs. dye concentration. Supporting electrolyte: phosphate buffer solution pH 5, accumulation time 180 s.

Under the selected optimal conditions the dependences I_pa_ = f (C_dye_) of one dye in the presence of another at a dye concentration-time of 180 s were obtained (Figure 10). The linearity of the dependences was observed in the concentration range 0.005–10.0 µM for SY and 0.02–7.5 µM for TZ in accordance with the linear regression Equations (14) and (15), respectively:

I_pa_(µA) = 136.25 C_SY_ (µM) + 1.05, R^2^ = 0.99 (14)

I_pa_(µA) = 32.34 C_TZ_ (µM) − 0.96, R^2^ = 0.99 (15)

The obtained LODs stood at 0.78 for SY and 8.2 nM for TZ.

The extension of the linear range in the area of high concentrations of the dyes with a shorter accumulation time on the electrode may save time and cost of the analysis when determining large concentrations of colorants. With a shorter duration of dye concentration on GrP/CP (60 s instead of 180 s), the upper limit of the linearity of the dependence I_pa_ = f(C_dye_) increases to 12 µM for SY and 25 µM for TZ. At 60 s accumulation time of the colorants on the GrP/CP electrode, the linear ranges are 0.02–12 µM for SY and 0.02–25 µM for TZ, and are described by linear regression Equations: I_pa_(µA) = 12.02 C_SY_ (µM) + 0.04 and I_pa_(µA) = 3.96 C_TZ_ (µM) − 0.82.

Table 2 presents the analytical characteristics of the developed sensor compared with the features of other existing sensors. As can be observed, the proposed sensor has similar or even better characteristics than other modified electrodes.

### 3.8. Interference

The findings of a study of the effect of some organic and non-organic interferents, present in beverages, on analytical responses of SY and TZ are demonstrated in Table 3.

### 3.9. Determination of SY and TZ in Real Samples

Real samples of alcoholic and non-alcoholic drinks containing SY and TZ both individually and jointly were analyzed, using the GrP/CP electrode by the standard addition method. The results of determining the content of SY and TZ in real samples are presented in Table 4. The accuracy of determination was evaluated by recovery tests. As can be seen in Table 4, the value of recovery is in the range from 96% to 104% and the relative standard deviation (RSD) does not exceed 0.072, confirming the accuracy and good reproducibility of the analysis results.

According to the obtained data, the levels of SY and TZ in the samples do not exceed 11.3 µM (5.1 mg L^−1^) and 10.3 µM (5.5 mg L^−1^), respectively. These values are significantly lower than the maximum permitted levels of these colorants in non-alcoholic/alcoholic drinks, i.e., 50/200 mg L^−1^ for SY and 100/200 mg L^−1^ for TZ, as recommended by the Joint FAO/WHO Expert Committee on Food Additives [9,10].

## 4. Conclusions

For the first time, an electrochemical sensor based on carbon paper was developed for the determination of SY and TZ. In comparison with other modern carbon materials (carbon nanotubes, graphene, carbon black), the strongest sensory response was obtained through volumetric modification of carbon paper with graphite powder. Modification of the carbon paper electrode has ensured almost twice as large an electroactive area and significant improvement in the charge transfer rate compared to a bare carbon paper electrode. Optimization of the volume of the modifier and registration conditions for differential pulse voltammograms has resulted in the best response of SY and TZ.

The parameters were calculated and the mechanism of colorant electrooxidation on the surface of the developed sensor was proposed. The sensor has high analytical characteristics, which include low detection limits at the nanomolar level (0.78 nM for SY and 8.2 nM for TZ); wide linearity ranges (three orders of magnitude); high selectivity and good reproducibility of the analytical signal. The special properties of the developed sensor are simplicity and the fabrication scale, which makes the cost of the sensor very low and enables it to be used as a one-time sensor. Excellent reproducibility of the analysis results allows us to recommend the developed sensor for express, highly sensitive and selective detection of SY and TZ in soft and hard drinks.

## Figures and Tables

**Table 1:** Characteristics of carbon materials used for studies.

Carbon Material	Characteristic
CP	d = 5–10 µm
	C > 95%
	surface density—30 gm^−2^
	surface electrical resistivity—8–10 Ω
MWCNTs	d = 110–170 nm
	l = 5–9 µm
	C > 90%
CB	20–25 nm
	C > 97%
	density 1.7–1.9 gcm^−3^
	surface area 30–300 m^2^g^−1^
GR	d = 5–10 µm
	h = 3–10 nm
	C ≥ 99%
GrP	d = 1–2 μm
	C ≥ 96% (Figure S1)

d—diameter; l—length; h—height; C—carbon weight content (%).

**Table 2:** Analytical characteristics and conditions of SY and TZ using different sensors.

Electrode	SY		TZ		pH	Method	t_acc_, s	E_acc_, V	Ref.
	LR, μM	LOD, nM	LR, μM	LOD, nM					
GO/MWCNTs/GCE	0.09–8	25	0.09–8	10	5	LSV	70	-	[26]
ERGO-AuNRs/GCE	0.01–3.0	2.4	0.03–6.0	8.6	6	DPV	300	0.3	[29]
ERGO/SPCE	0.01–20	5	0.02–20	4.5	6	DPV	180	-	[25]
Fe_3_O_4_@SiO_2_/MWCNTs/CPE	0.5–20, 20–100	50	0.5–14, 14–100	40 70	6	SWV	180	0.2	[34]
PLC/Ag/GCE	0.5–10, 10–300	75	0.75–75, 75–750	250	4.5	DPV	-	-	[61]
ZnO/Cysteic acid	0.1–3.0	30	0.07–1.86	10	5	DPV	120	−0.05	[33]
rGO/NiBTC/SPCE	0.05–5.0	25	0.075–5.0	50	8	DPV	360	0.1	[62]
ZnCrFeO_4_/CPE	0.05–19	2	0.07–47.5	10	6	DPV	-	-	[59]
AuNPs/PDDA/Gr/GCE	0.006–5.0	2	0.008–3.0	2.5	7	DPV	360	-	[28]
GCILE	0.02–5	10	0.5–15	100	7	DPV	150	0.5	[63]
GrP/CP	0.005–1.0	0.78	0.02–7.5	8.2	5	DPV	180	0	This work

LR—linear range; LOD—limit of detection; LSV—linear sweep voltammetry; DPV—differential pulse voltammetry; SWV—square wave voltammetry. GO—graphene oxide; ERGO—electrochemically reduced graphene oxide; AuNRs—gold nanorods; PLC—poly(L-cystein); rGO—reduced graphene oxide; AuNPs—gold nanoparticles; PDDA—poly(diallyldimethylammonium chloride); GCILE—1-butylpyridinium hexafluorophosphate@glassy carbon microspheres composites electrode.

**Table 3:** The effect of various interferents on the simultaneous responses of SY and TZ (each 1 μM).

Interferent	Excess	Response Change, %	
		SY	TZ
Taurine	200	−4	−3
Ascorbic acid	300	−5	−2
Caffeine	300	−3	−6
Glucose	400	−2	0
Tartaric acid	400	−6	−3
Sodium citrate	400	0	−7
Ammonium chloride	800	−4	0
Sodium carbonate	1000	0	0
Calcium Chloride	1000	0	0

It is apparent from the data in this table that the applied volumes of the interferents do not affect the analytical responses of SY and TZ. In addition, it was found that in practical terms the presence of 20% ethanol has no impact on the signals of 1 µM SY (−6%) and 1 µM TZ (+1%).

**Table 4:** The results of SY and TZ determination in beverages (n = 5, *p* = 0.95).

Sample	Analyte	Detected, μM	RSD, %	Added, μM	Found, μM	RSD, %	Recovery, %
Alcoholic drink (beer)	SY	1.13 ± 0.1	5.1	1	2.09 ± 0.07	1.4	96
Non-alcoholic carbonated drink	TZ	10.3 ± 1.7	3.7	10	20.4 ± 1.4	2.8	101
Non-alcoholic energy drink	SY	11.3 ± 1.6	5.7	10	21.4 ± 2.0	3.8	101
	TZ	6.1 ± 1.1	7.1	5	11.3 ± 1.2	3.2	104
Alcoholic drink (liqueur)	SY	0.74 ± 0.04	2.3	0.5	1.24 ± 0.06	2.0	100
	TZ	0.046 ± 0.005	4.3	0.05	0.094 ± 0.008	3.9	96
Carbonated drink	SY	0.35 ± 0.04	4.2	0.2	0.55 ± 0.03	2.1	100
	TZ	0.103 ± 0.015	6.3	0.1	0.200 ± 0.009	5.2	97

## Data Availability

Not applicable.

## Supplementary Materials

The following supporting information can be downloaded at: https://www.mdpi.com/article/10.3390/s22114092/s1, Figure S1: SEM image (a) and EDX spectra of graphite powder (b); Figure S2: Cyclic voltammograms of CP (a) and GrP/CP (b) electrodes in the presence of 1.0 mM K_3_[Fe(CN)_6_] solution in 0.1 M KCl at various scan rates: 50, 75, 100, 125, 150, 200, 250, 300 mV s^−1^; Figure S3: Choice of optimal conditions for colorant accumulation on the GrP/CP and recording of differential pulse voltammograms: potential of accumulation (a), modulation amplitude (b), step potential (c) and modulation time (d).
