# Supplementary material for: An Electrochemical Sensor Based on Carbon Paper Modified with Graphite Powder for Sensitive Determination of Sunset Yellow and Tartrazine in Drinks
Source: Sensors (Basel). 2022 May 27;22(11):4092. doi: 10.3390/s22114092 (PMC9185310; doi:10.3390/s22114092)
Supplement: Supplementary file 1 [file sensors-22-04092-s001.zip › sensors-1727362-supplementary.pdf]

Supplementary Materials

An electrochemical sensor based on carbon paper modified by graphite powder for sensitive determination of Sunset Yellow and Tartrazine in drinks

Natalia Yu. Stozhko*, Ekaterina I. Khamzina, Mariya A. Bukharinova, Aleksey V. Tarasov

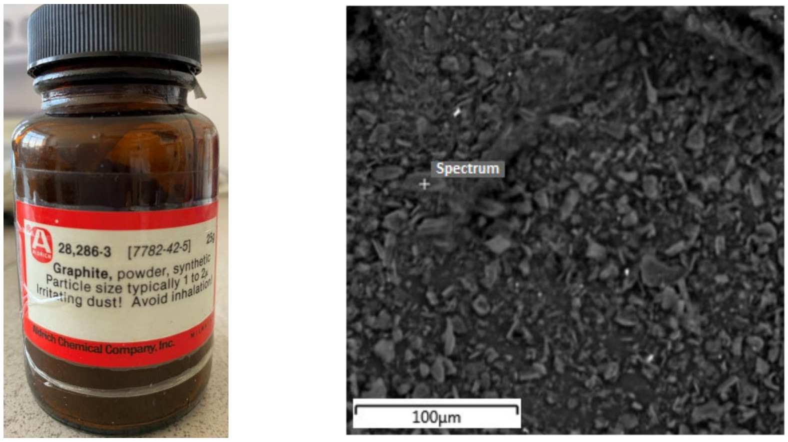

(a)

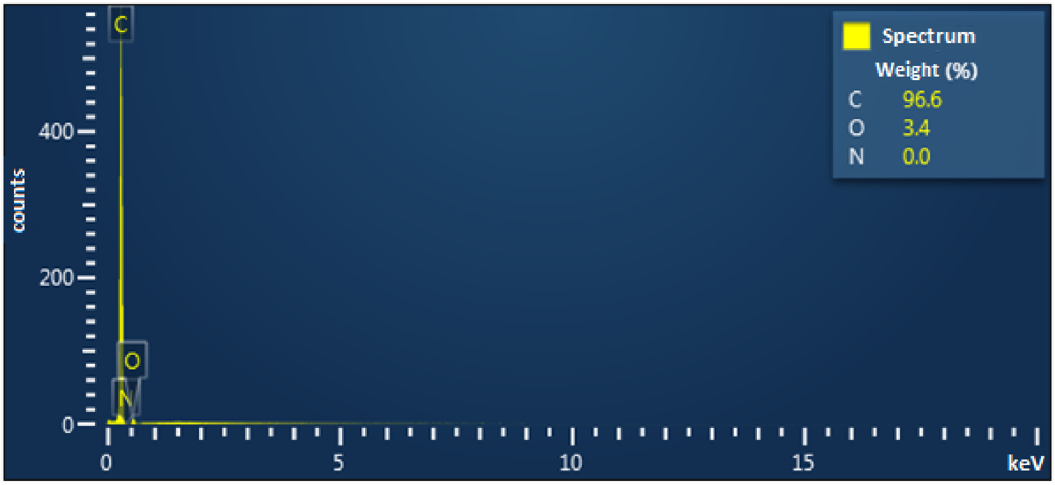

(b)

Figure S1. SEM image (**a**) and EDX spectra of graphite powder (**b**).

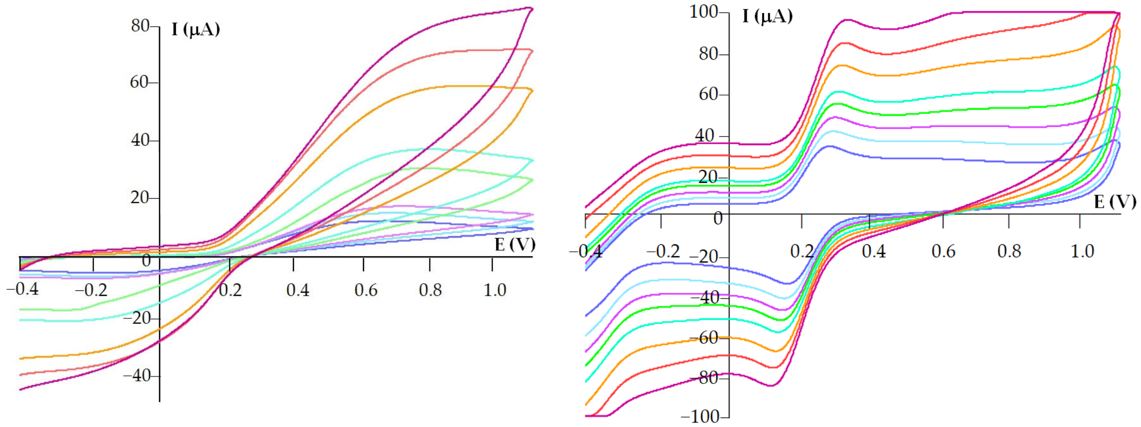

(a) (b)

Figure S2. Cyclic voltammograms of CP (**a**) and GrP/CP (**b**) electrodes in the presence of 1.0 mM $K_3[Fe(CN)_6]$ solution in 0.1 M KCl at various scan rates: 50, 75, 100, 125, 150, 200, 250, 300 mV s^{-1}.

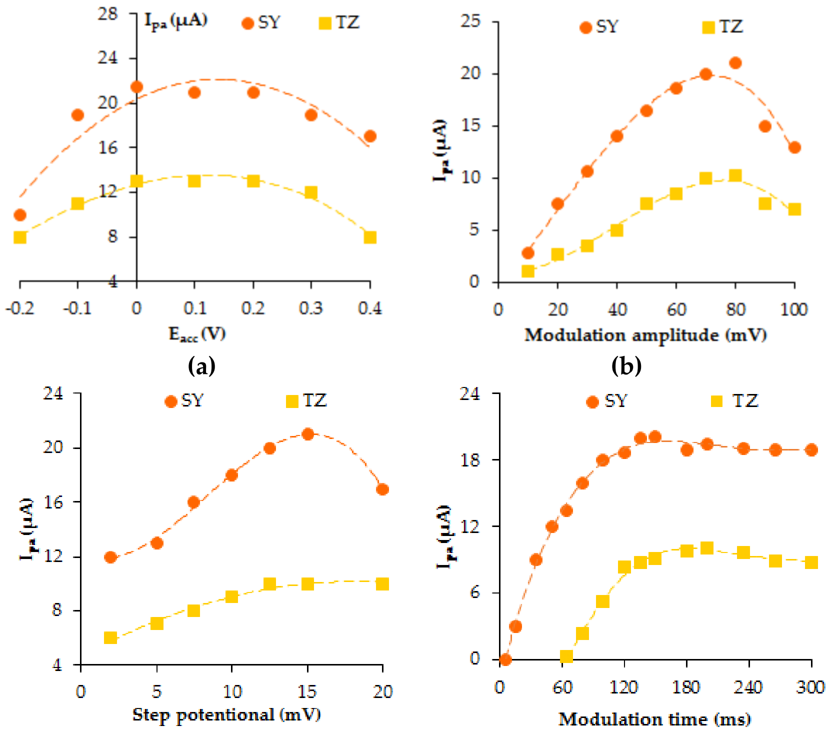

(c) (d)

Figure S3. Choice of optimal conditions for colorants accumulation on the GrP/CP electrode and recording of differential pulse voltammograms: potential of accumulation (E_{acc}) (**a**), modulation amplitude (**b**), step potential (**c**) and modulation time (**d**).
